# Methods of space maintenance for premature loss of a primary molar: a review

**DOI:** 10.1007/s40368-018-0357-5

**Published:** 2018-09-05

**Authors:** A. J. Ahmad, S. Parekh, P. F. Ashley

**Affiliations:** Department of Paediatric Dentistry, Eastman Dental Institute, Eastman Dental Hospital, University College London, 256 Gray’s Inn Road, London, WC1X 8LD UK

**Keywords:** Space maintenance, Space maintainer, Deciduous molar, Primary molar, Tooth loss

## Abstract

**Aim:**

This critical appraisal attempts to answer the question: What is the best method of space maintenance (SM) following premature loss of a primary molar in children under 12 years old?

**Methods:**

A search to identify studies relevant to the PICO was conducted. Single case reports and studies prior to 1986 were excluded. The principles of GRADE were followed to appraise the evidence.

**Results:**

20 studies were identified, which evaluated 2265 space maintainers (SMs). Two studies were graded high quality, four moderate, eight low, and six very low. All studies reported on longevity outcomes and most on adverse effects.

**Conclusions:**

There was no strong evidence favouring a particular SM, the following recommendations were made: (a) strong recommendations: In cases where rubber dam cannot be used clinicians should not use Glass Fibre Reinforced Composite Resin (GFRCR) SMs. (b) Weak recommendations: Crown and Loop SMs are recommended for loss of primary first molars; GFRCR SMs (placed under rubber dam) are recommended for loss of primary second molars. Bilateral SMs may have questionable efficacy and their use where there is loss of multiple molars in the same quadrant should be weighed against the risk of unwanted tooth movements, loss of a removable SM or no space maintenance at all.

## Introduction and aim

The phenomenon of space loss following premature loss of a primary molar was first described in 1887 (Davenport 1887). Adverse effects of space loss are reported to include; crowding of the dental arch, ectopic eruption and impaction of the permanent tooth, tipping of the first permanent molar, crossbite formation and centre line discrepancies (Richardson 1965; Clinch and Healy 1959). Evidence for and against the use of space maintainers to avoid these effects is weak (Laing et al. 2009), yet they are commonly used by clinicians worldwide.

SMs can be fixed or removable, unilateral or bilateral. Fixed unilateral SMs include the Band and Loop (B&L), Crown and Loop (C&L), Direct Bonded (DB), Glass Fibre Reinforced Composite Resin (GFRCR) and Distal End Shoe (DES). Fixed bilateral SMs include the Lower Lingual Arch (LLA), Nance and Transpalatal Arch (TPA).

The aim of this review was to critically appraise the evidence evaluating different types of SMs in order to identify the best methods of space maintenance following premature loss of a primary molar.

## Materials and methods

### The review question

The review question was developed using a PICO structure:

What is the best method of space maintenance following early loss of a primary first or second molar in children under 12 years old?

In order to appraise the ‘best method’, seven areas of assessment of SMs were identified in as follows: Achievement of Clinical Goals (examples could include prevention of a malocclusion, reduction in orthodontic need), Efficacy in preventing space loss, assessment of Adverse Effects, Patient Reported Outcomes Measures (PROMs), Longevity, Practicality and Costs of Delivery. Outcomes relating to these seven categories were included in the study.

### Search strategy

Ovid Medline was searched to combine studies related to the MeSH heading of ‘space maintenance’ or the terms ‘band and loop’, ‘crown and loop’, ‘nance’, ‘transpalatal’, ‘lingual arch’, ‘distal end shoe’, ‘space hold*’ or ‘space maint*’ together with the MeSH headings of ‘deciduous tooth’, ‘tooth loss’ or ‘molar’ or the terms: ‘molar’ or ‘tooth extraction’. The search results were limited to include all child (0–18 years) related results. A similar strategy was repeated using Embase and the Cochrane library.

**Fig. 1:**
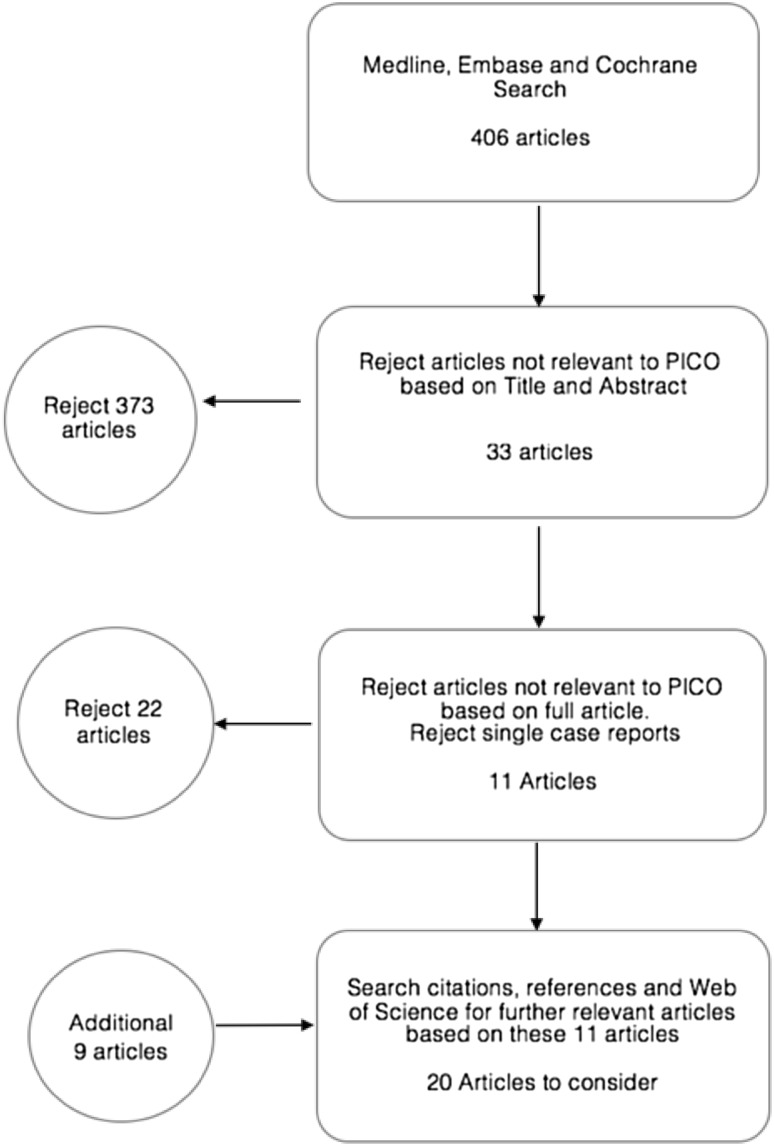
Summary of search strategy with inclusions and exclusions

The search strategy is summarised in Fig. 1. The Inclusion and exclusion criteria are summarised in Table 1.

**Table 1:** Inclusion and exclusion criteria

Inclusion criteria	Primary studies A SM is used following premature loss of a primary molar in a child < 12 years Outcomes for the SM are reported Published after 1986
Exclusion criteria	Single case reports

### Appraising the evidence

The studies were individually assessed as being High, Moderate, Low or Very Low quality according to the principles of grading the quality of evidence outlined in ‘Grading of Recommendations Assessment, Development and Evaluation’ (GRADE) (Guyatt et al. 2010), The full GRADE methodology was not implemented.

GFRCR SMs placed with and without rubber dam differ in their methods significantly. Moisture control for resin bonding specifically in younger children is likely to be affected when bonding without rubber dam. This in turn is likely to affect the bond strengths and longevity of the SMs, for this reason and to eliminate the inconsistencies this may cause, the outcomes were reported separately for GFRCR SMs placed with rubber dam and GFRCR SMs without rubber dam.

## Results

A total of 406 articles were identified as potentially relevant from the database searches, with 373 articles excluded after applying the PICO based inclusion criteria to the titles and abstracts. The same inclusion criteria applied to the full article text excluded another 11 articles, with 11 further exclusions based on study design. A Web of Science search of the 11 remaining articles and a check of their citations and references led to inclusion of an additional nine articles.

Twenty relevant articles were appraised and graded for the quality of the study; are summarised in Table 2. Reporting of outcomes according to the seven point assessment criteria is summarised in Table 3.

**Table 2:** Summary of included articles and grading

Study	Description	Justification for Grading	Grade
Qudeimat and Sasa (2015)	Controlled Clinical trial Compared B&L and C&L SMs 52 months length Loss of primary first molars	Well conducted study, risk of bias from non randomised allocation, however this was due to ethical reasons and was in favour of the control group. The very large and significant difference in the magnitude of effect of the study increased the quality grading to ‘High’	High
Garg et al. (2014)	Split Mouth RCT Compared GFRCR placed with rubber dam and B&L SMs 6 month observation Loss of a primary first molar	Well reported study, however risk of bias through poor description of randomisation method and risk of imprecision through short length of observation	Moderate
Gulec et al. (2014)	Case Series of a commercially available DB SM (the E-Z SM) used following loss of one or two primary molars 20 month observation	Well reported, limited in quality due to nature of study	Low
Nidhi et al. (2012)	Split Mouth RCT Compared GFRCR placed with rubber dam and B&L SMs 5 month observation Loss of a primary first or second molar	No information on the method of randomisation and short observation period therefore downgraded due to risk of bias and imprecision	Moderate
Tunc et al. (2012)	RCT Compared B&L, DB and GFRCR SMs placed without Rubber Dam 12 month observation Loss of primary first or second molar	Method of randomisation not described, small sample sizes, confidence intervals not provided for findings, therefore downgraded to Moderate	Moderate
Owais et al. (2011)	RCT Compared space changes between LLAs constructed with 0.9 mm and 1.25 mm wire and a control group with no SM Loss of one or both lower primary second molars after eruption of permanent incisors	Well randomised, controlled and fully reported, findings consistent with other studies, directly relevant to PICO	High
Sasa et al. (2009)	Uncontrolled prospective study of B&L SMs 40 month follow up Loss of primary first molars	Well conducted, well reported, long observation period, limited on quality due to lack of a control group	Low
Subaramaniam et al. (2008)	Split mouth RCT Compared GFRCR placed with rubber dam and B&L SMs 12 month observation Loss of primary first molars	Method of randomisation not described, some reporting does not reflect the method therefore downgraded to moderate	Moderate
Fathian et al. (2007)	Retrospective reporting on treatment notes for all B&L, Nance and LLA SMs placed by a single practitioner over a 7 year period Loss of a first or second primary molar. Failed appliances re-included as new appliances	Directly relevant to the PICO however limited in quality due to inherent low quality of observational studies	Low
Moore and Kennedy (2006)	Retrospective reporting on treatment notes for Nance and LLA SMs placed by two different practitioners over 7 year period SMs used to hold the leeway space, with and without premature loss of a primary molar	Some indirectness although this was not severe, limited in quality due to inherent low quality of observational studies	Low
Yilmaz et al. (2006)	Case studies of DB SM bonded with 12 month follow up. Window cut through a PMC exposing buccal enamel surface to which a DB SM was bonded. Premature loss of primary first or second molars	No control group, no criteria for assessing failures, and incomplete data reported; severe risk of bias so downgraded	Very low
Tulunoglu et al. (2005)	Described itself as a retrospective study, however the description of its method is prospective Unable to Distinguish types of SMs used	Very poorly reported, Method unclear and vague, high ‘lost to follow up rate’, severe risk of bias, findings were not included in the outcome analysis	Very low
Kargul et al. (2005)	Case Studies of GFRCR SMs placed without rubber dam 12 month follow up	Very little information and lack of data presented to justify findings or conclusions; severe risk of bias and imprecision	Very low
Kirzioglu and Erturk (2004)	Case Studies of GFRCR SMs placed without rubber dam 24 month observation period	Limited in quality due to inherent low quality of observational studies, some indirectness however data presented clearly to identify those relevant to PICO Some incomplete outcome reporting but not at severe risk bias so not downgraded	Low
Simsek et al. (2004)	Case Studies of DB SMs 12–18 month follow up	No clear inclusion criteria or predefined failure criteria and method unclear, no data presented to substantiate conclusions, failures of composite bonding were still included as successes; severe risk of bias, inconsistency and imprecision	Very low
Rajab (2002)	Prospective Cohort study 60 months duration Reporting on B&L, Nance, LLA and Removable SMs	Some indirectness, unclear inclusion criteria, no confidence intervals provided and high loss to follow up (19.9%); therefore at risk of imprecision	Low
Brill (2002)	Case Studies over a 6 year period of crown retained DES SM Loss of a primary molar with no tooth distal to the space (one case after extraction of a permanent first molar)	Risk of bias as one practitioner assessing, treating and publishing the report, lack of a control group and severe risk of bias, 43% still in use at the time of publication, no minimum observation period provided; severe risk of imprecision	Very low
Qudeimat and Fayle (1998)	Retrospective reporting on treatment notes of all patients who had SMs during a five year period at a dental hospital, reported on B&L, Nance, LLA and Removable SMs	Low quality due to study design, not downgraded further although some risk of inconsistency and indirectness as SMs not used exclusively for primary molar loss	Low
Baroni et al. (1994)	Described as an observational study of 53 months B&L SMs, Nance SMS and LLA SMs	Incomplete reporting, no inclusion criteria and insufficient description of the method of this study to ascertain if it was retrospective or prospective, method unclear and vague; severe risk of bias and imprecision	Very low
Santos et al. (1993)	Case series of DB SMs Six months observation Loss of primary first or second molar	Limited quality due to study type, some lack of detail in reporting however not severe risk of bias so not downgraded	Low

**Table 3:** Reporting of outcomes according to the seven point assessment criteria

Study	Outcomes reported						
	Clinical goals	Efficacy	Adverse effects	PROMs	Longevity	Practicality	Costs
Qudeimat and Sasa (2015)			✓		✓		
Garg et al. (2014)			✓	✓	✓	✓	
Gulec et al. (2014)		✓	✓		✓	✓	
Nidhi et al. (2012)			✓		✓		
Tunc et al. (2012)			✓		✓		
Owais et al. (2011)		✓			✓		
Sasa et al. (2009)			✓		✓		
Subaramaniam et al. (2008)			✓		✓	✓	
Fathian et al. (2007)			✓		✓		
Moore and Kennedy (2006)			✓		✓		
Yilmaz et al. (2006)		✓	✓		✓		
Tulunoglu et al. (2005)					✓		
Kargul et al. (2005)			✓		✓		
Kirzioglu and Erturk (2004)			✓	✓	✓	✓	
Simsek et al. (2004)		✓			✓		
Rajab (2002)			✓		✓		
Brill (2002)					✓		✓
Qudeimat and Fayle (1998)			✓		✓		
Baroni et al. (1994)			✓		✓		
Santos et al. (1993)		✓	✓	✓	✓		
Total studies reporting outcome	0	5	16	3	20	4	1

No studies reported outcomes relating to achievement of the clinical goals of a SM. All studies reported longevity outcomes, the qualitative longevity outcomes are summarised in Table 4, other findings are summarised by the type of SM.

**Table 4:** Longevity outcomes of included articles

Study	SM assessed (n)	Summary of longevity findings	
		Failure rates (over × months)	Mean survival length in months
High quality studies			
Qudeimat and Sasa (2015)**	B&L (18)	33% (12)* 67% (36)* 83% (52)	18.78 SE = 3.10 95% CI (12.69–24.86)
	C&L (18)	6% (12)* 17% (36)* 22% (52)	40.4 SE = 2.56 95% CI (35.36–45.42)
Owais et al. (2011)**	LLA (20) LLA (24) No LLA (23)	Failed appliances re-included	This paper evaluated the efficacy of the SMs and did not calculate survival lengths
Moderate quality studies			
Garg et al. (2014)**	B&L (30)	64% (6)	
	GFRCR (30)	36.7% (6)	
Nidhi et al. (2012)**	B&L (20)	37% (5)	
	GFRCR (20)	21% (5)	
Tunc et al. (2012)**	B&L (10)	0% (6) 10% (9) 10% (12)	11.20
	GFRCR No Rubber Dam (10)	60% (6) 80% (9) 80% (12)	6.7
	DB (10)	30% (6) 60% (9) 60% (12)	9.20
Subaranium et al. (2008)**	B&L (30)	57% (6) 67% (12)	
	GFRCR (30)	33% (6) 47% (12)	
Low quality studies			
Gulec et al. (2014)	E-Z (41)	15% (12)	7^!^ Mean survival time provided is lower than each group's survival time, possible error in results
Sasa et al. (2009)	B&L (40)	13% (12)* 46% (36)* 59% (40)*	19.9 (median) SE = 8.1 95% CI (4.1–35.7)
Fathian et al. (2007)	B&L (112)	Failed appliances re-included	Maxillary 26.0 SD = 17.3 Mandibular 27.7 SD = 14.4
	Nance (69)	Failed appliances re-included	25.5 SD = 14.7
	LLA (142)	Failed appliances re-included	26.9 SD = 14.4
Moore and Kennedy (2006)	Nance (205)	Failed appliances re-included	22.7 SD: 12.2
	LLA (207)	Failed appliances re-included	19.9 SD = 11.0
Kirzioglu and Ertuk (2004)	GFRCR No Rubber Dam (31)	94% (12)	
Rajab (2002)	B&L (171)	35% (60)	20 (median)
	Nance (69)	20% (60)	24 (median)
	LLA (115)	57% (60)	14 (median)
	Removable (32)	26% (60)	
Qudeimat and Fayle (1998)	B&L (81)	Failed appliances re-included	13 (median)
	Nance (30)	Failed appliances re-included	6 (median)
	LLA (71)	Failed appliances re-included	4 (median)
	Removable (82)	Failed appliances re-included	
Santos et al. (1993)	DB (60) (chair side fabrication)	2% (4) 8% (6)	
Very low quality studies			
Yilmaz et al. (2006)	DB to Open Faced Crowns (23)	0% (12)^!^	
Kargul et al. (2005)	GFRCR No rubber Dam (23)	57% (12)^!^	5^!^ Survival times only calculated for failed SMs
Simsek et al. (2004)	DB (74)	5% (16)^!^	
Brill (2002)	DES (190)	12% (unknown)^!^	
Baroni et al. (1994)	B&L (33)	30% (36)^!^	
	Nance (19)	30% (36)^!^	
	LLA (36)	60% (36)^!^	
Tulunoglu et al. (2005)	Unable to distinguish types of SMs used		

*B&L* band and loop, *C&L* crown and loop, *DB* direct bonded, *DES* distal end shoe, *GFRCR* glass fiber reinforced composite resin, *LLA* lower lingual arch

*Values calculated from data provided in the study

**This study was designed in a manner that the SMs within this study can be directly compared

^!^Very low confidence in this finding

Where studies provided success rates, these were converted to failure rates as (100% − success rate). Where failure rates were not directly calculated by the authors and the raw data was available these were calculated to exclude those lost to follow up (100 × failed appliances/(total appliances − lost to follow up)). Failure rates calculated from raw data and not directly provided by the study have been indicated with an *.

Results which have a very low confidence in their reliability are highlighted with !.  Failure rates provided by studies which re-included failed appliances have been omitted.

The heterogeneity of studies and variation between methods of calculating failure and survival data prevented meta analysis to provide estimates of survival lengths or failure rates. The only SMs assessed within studies, were those from Qudeimat and Sasa (2015) Garg et al. (2014), Nidhi et al. (2012), Owais et al. (2011) and Subaranium et al. (2008).  Therefore these are the only studies where direct comparisons between SMs could be made.

The efficacy of the SM in preventing loss of the primary molar space was reported in 5 studies. SMs with a rigid component in the primary molar space will prevent space loss as long as the SM is retained. Therefore, the longevity outcomes were used to approximate to the efficacy of the following SMS, B&L, C&L, GFRCR, and Removable SMs.

### Band and Loop (B&L) Space Maintainers

Ten studies (one high, four moderate, four low and one very low quality) evaluated B&L SMs with a total sample size of 545 SMs and a maximum follow-up period of 52 months. Cement loss or decementation were cited as the most common cause of failures in all studies. Failures resulting from soft tissue lesions were noted in many studies, and was the cause of up to 14% of all failures seen by Nidhi et al. (2012). Some of these were due to the metal components impinging on the soft tissues, either through solder breakage, or slippage of the band secondary to cement loss. If this occurred and was not managed soon there is potential for the metal component to become embedded in the soft tissues.

### Glass fibre reinforced (GFRCR) space maintainers

Six studies (four moderate, one low and one very low quality) evaluated GFRCR SMs with a total sample size of 144 SMs and a maximum follow-up period of 12 months. Failure of the composite to enamel bond was the main cause of failure in all studies. The average times for placement of band and loop SMs was ‘in excess of 30 min’ whilst GFRCR SMs, required an average chair side of 1–15 min (Garg et al. 2014). This is described as being significant however no p value was provided. Garg et al. (2014), was the only study to include Patient Reported Outcome Measures (PROMs) in their method. The Wong-Baker scale (Wong-Baker 2015) was used to record discomfort/preference for B&L SMs and for the GFRCR SM. The results were significantly different (p < 0.001) with B&L SM scoring an average of 6.40 (Hurts even more) and GFRCR scoring an average of 1.33 (No Hurt). The studies assessing these SMs were short in length and their use beyond a period of 12 months was not assessed in any study. The positioning of the GFRCR band and the effect this has on failure rates was not assessed.

### Direct bonded (DB) space maintainers

Five studies (one moderate, two low and two very low quality) evaluated DB SMs with a total sample size of 208 SMs and a maximum follow-up period of 12 months. The most common cause of failure in all studies was failure of the composite-enamel bond. DB SMs were shown to be effective in preventing space loss in the three studies which measured efficacy (Santos et al. 1993; Simsek et al. 2004; Yilmaz et al. 2006). This is in agreement with a previous study by Swaine and Wright (1976) which also found direct bonded SMs to be effective in preventing space loss and rotations of teeth.

Gulec et al. (2014), presented case studies of a commercially available, prefabricated and adjustable DB SM, the E-Z SM, this type of DB SM was found to be associated with an increase in gingival inflammation index but had no significant adverse effects. The average chair-side time for placement of the DB E-Z SM was recorded as15.5 min, there was no control in this study to compare this value against.

### Crown and loop (C&L) space maintainers

One high quality study evaluated C&L SMs with a sample size of 18 SMs and 52 months follow up (Qudeimat and Sasa 2015). C&L SMs have not been a popular choice of SM. During a five year period in a UK university based hospital only one SM out of a total of 301 SMs fitted was a C&L SM (Qudeimat and Fayle 1998). Possible reasons given for the lack of use of C&L SMs were the need for a temporary crown during fabrication of the C&L device, and concerns about replacing the crown in the event of failures. Qudeimat and Sasa (2015), described a method without use of a temporary crown, and in the event of failures they described removing the loop from failed C&L SMs and converting these to conventional B&L SMs by placing the bands over the crowns. The authors claim that the methods they have described remove the reasons given for their lack of popularity. The most common cause of failure was solder breakage, with none attributed to cement failure.

### Distal end shoe (DES) space maintainers

One very low quality study evaluated a form of DES SM with a sample size of 190 SM, the follow-up period was unclear. Brill (2002) describes the procedure for the chairside fabricated DES as being easy to perform and economical by way of excluding the need for a second appointment and laboratory expenditures. However, these statements were unsubstantiated. The chair-side assembly of the distal end shoe immediately following extraction, followed by soldering the distal end shoe to a prefabricated crown gave the impression that it could be a technically difficult and long procedure to perform. This, combined with the need for soldering equipment to be available in the surgery, could make this procedure costly and difficult. There was no formal assessment of gingival health around the subgingival component of the appliance. Similar to the C&L SM the most common cause of failure was solder breakage.

### Lower lingual arch (LLA) space maintainers

Six studies (one high, four low and one very low quality) evaluated LLA SMs with a total sample size of 615 SMs, the follow-up period was unclear. The most common adverse effects reported with these appliances were interference with eruption of permanent teeth (all instances were caused if the appliance was placed before eruption of the permanent incisors) and soft tissue lesions. All studies reported ‘cement loss’ as the main cause of failures.

An RCT found that whilst LLA appliances preserved arch length, the primary molar space was reduced. Preservation of arch length was achieved at the expense of proclination of the lower incisors, and an increase in the inter-canine width (Owais et al. 2011).

### Nance SMs

Five observational studies (four low and one very low quality) evaluated Nance SMs with a total sample size of 392 SMs, the follow-up length was difficult to ascertain. Rajab (2002) and Baroni (1994) reported a high proportion of failures due to soft tissue lesions, these were unspecified and may be related to the acrylic button which contacts the anterior palate. All studies reported ‘cement loss’ as the main cause of failures. No studies reported on the efficacy of the SM in achieving antero-posterior space maintenance.

### Transpalatal arch (TPA) SMs

No studies reported on TPA SMs. TPA and Nance appliances, have similarities in design and both aim to prevent space loss by preventing mesial movement, tipping and rotation of the first permanent molars. Kupietzky and Tal (2007) presented an opinion paper suggesting TPAs should be used in preference to Nance appliances, raising concerns about the soft tissue irritation from the acrylic button on the Nance appliance as a reason.

Stivaros et al. (2010) conducted a RCT which compared the efficacy of Nance and TPA appliances during fixed orthodontic appliance therapy. This study was not included in the present review as it is not relevant to the PICO, however it provides indirect evidence which is useful in evaluating and comparing Nance and TPA SMs. It was found that although there was some mesial drift and tipping of the first permanent molars with both appliances there was no significant difference between the magnitude of the movements between the two appliances (p > 0.05) however there was significantly more patient discomfort reported with the Nance appliance (p = 0.001) and therefore TPA SMs may be preferable to Nance SMs.

### Removable SMs

Two low quality observational studies evaluated 114 removable SMs between them. The most common cause of failure for the removable SMs was ‘complete loss’.

## Discussion

There was no strong evidence favouring a particular method of space maintenance. Evidence to evaluate achievement of clinical goals, patient preference and costs of delivery was poor and recommendations cannot be made based on these outcomes. In assessing the best method of space maintenance we rely heavily on longevity and efficacy outcomes. Failure rates varied largely between studies, with the exception of C&L SMs, all estimates of the mean/median survival times of other SMs did not exceed two years. It is reasonable to expect that SMs may need replacement or repair during treatment. Clinicians should therefore also take into account the ease of repair, maintenance, and risk of adverse effects when selecting a method of space maintenance.

Cement loss or decementation was the most common cause of failure of all band retained SMs. Crown retained SMs did not exhibit the same failures and may eliminate the problem of cement failures seen with band retained SMs; this may also account for the superior longevity outcomes of C&L SMs. Although the Qudeimat and Sasa’s study (2015) was graded a high quality study the longevity findings were based on only one study, and therefore the evidence for the recommendation of C&L SMs is weak.

GFRCR SMs placed under rubber dam showed comparable or better longevity outcomes to B&L SMs in studies which compared them directly. GFRCR SMs benefit from much shorter procedural times, single visit placements and a relatively simple procedure for repairs and replacements. The studies evaluating GFRCRs were very short in length, and this would ordinarily prevent even a weak recommendation for their use. However, as the evidence for all other existing SMs is also weak and there may be less incidence of adverse effects compared to the more widely used metal SMs a weak recommendation is being made for the use of these SMs. Their use should be limited to loss of a single tooth, bounded by abutment teeth with sound enamel surfaces for bonding and only where rubber dam can be used in their placement. The use of a coloured composite may facilitate safe removal of the SM. If these SMs are used they should be followed-up closely and alternative methods used in the case of repeat failures. GFRCRs placed without rubber dam showed very high failure rates, Kirzigolu and Erturk (2004) recommended that these SMs be used for short periods only. A strong recommendation is made to avoid the use of GFRCR SMs when a rubber dam cannot be used.

There was weak evidence that LLAs may have poor efficacy in maintaining the primary molar space.  These findings are in agreement with Rebellato et al. (1997) who also reported that LLAs can increase the total arch length as a result of distal movement of the molars and lower incisor proclination.  There were also poor longevity outcomes in all but two studies, therefore a weak recommendation is made to avoid the use of LLA SMs if other SMs can be used.

TPAs and Nance appliances may prevent molar movement but have no effect on space loss from the distal movement of canines into the primary molar space. The lack of direct evidence of efficacy of TPAs and Nance appliances combined with loss of the primary molar space with LLA SMs (Owais et al. 2011) brings into question the efficacy of bilateral SMs in preventing loss of the primary molar space. Therefore, their use should be balanced with the lack of evidence for their efficacy and risk of loss of primary molar space.

TPA appliances were favoured over Nance appliance in an opinion paper by Kupietzky (2007) and indirect evidence from a high quality RCT conducted by Stivaros et al. (2010) suggested they may cause less patient discomfort and be more effective in space maintenance. As this evidence is indirect, only a weak recommendation for TPA appliances in preference over Nance appliances can be made.

In the case where the tooth distal to the primary molar space is unerupted (distal free end) only one type of SM was evaluated, however the study was assessed as being at severe risk of bias, and of very low quality.  Therefore no recommendations could be made for or against a SM to be used in this scenario.

## Conclusions

C&L SMs have the best longevity and GFRCR SMs may be a longer lasting and safer alternative to B&L SMs. C&L SMs are recommended for loss of a primary first molar and GFRCR SMs (placed under rubber dam) are recommended for loss of a primary second molar.

Where there is loss of multiple molars in the same quadrant, the options for SMs are more limited.  Bilateral SMs may have questionable efficacy and their use should be weighed against the risk of unwanted tooth movements, loss of a removable SM, or no space maintenance at all.

Brill (2002) states that the success of an appliance lies on whether the appliance achieved what it was designed to do for the patient, even if it needed service or repair during the course of treatment. With this in mind clinicians are advised to select SMs with the expectation that the period of space maintenance will likely require replacements, repairs and perhaps even a number of different types of SMs until the end point of therapy.
